# A series of compact rejection filters based on the interaction between spoof SPPs and CSRRs

**DOI:** 10.1038/srep28256

**Published:** 2016-06-21

**Authors:** Qian Zhang, Hao Chi Zhang, Jia Yuan Yin, Bai Cao Pan, Tie Jun Cui

**Affiliations:** 1 State Key Laboratory of Millimeter Waves, Southeast University, Nanjing 210096, China; 2 Synergetic Innovation Center of Wireless Communication Technology, Southeast University, Nanjing 210096, China; 3 Cooperative Innovation Centre of Terahertz Science, No.4, Section 2, North Jianshe Road, Chengdu 610054, China

## Abstract

We propose a method to synthesize several band-rejection filters by etching split-ring resonators (SRRs) on the transmission line for spoof surface plasmon polaritons (SPPs), which is made of double-side or single-side corrugated metal strips. From dispersion relations, the corrugated strips can support spoof SPP modes when the operating frequency is less than the cutoff frequency. The electric field component perpendicular to the strip surface of the SPP modes can excite the complementary SRRs (CSRRs), leading to resonant modes preventing the SPP propagation near the resonant frequencies. Using this principle, single-frequency rejection filters, double-frequency rejection filters, and broad band-stop filters with bandwidth of 1.5 GHz have been designed and fabricated using the single- and/or double-side corrugated strips. Both measured results and numerical simulations demonstrate the excellent filtering characteristics of all design, which are in good agreements. The isolation of all filters can be less than −20 dB, and even reach to −38 dB at rejection frequencies. The proposed rejection and stop-band filters give important potentials to develop integrated plasmonic functional devices and circuits at microwave and terahertz frequencies.

Propagating surface plasmon polaritons (SPPs) are tightly fettered on the interface of metal and dielectric at the optical frequency^1^, behaving good performance for SPP transmission in the direction parallel to the metal surface with exponential decay in the direction normal to the surface^2^,^3^. Many scientists have devoted to research SPPs for the advantage of strong local-field enhancement and breaking the diffraction limit as propagating surface waves^4^,^5^. The main reason for the formation of the optical SPPs is the manifestation of the metallic negative permittivity at those frequencies^6^. However, in virtue of perfect electric conductors of the metal at lower frequencies (far infrared, terahertz and microwave bands), the plasmonic waves cannot be efficiently confined to the metal^7^.

To overcome this limitation, the plasmonic metamaterials of corrugated metal structures was proposed to excite the so-called spoof SPP modes, which have similar dispersion relations and spatial confinements to SPPs in the optical region^8^. Since then, there have been periodic arrays enchased with slits, holes or blocks of one- or two-dimensional structures reported for propping spoof SPP modes up availably^9^,^10^,^11^,^12^. The hybrid circuits combining traditional transmission line and ultrathin corrugated metallic strips have made the conversion and transition between the spatial guided waves and spoof SPPs possible^12^,^13^,^14^. In the meantime, the wide-band filters^15^,^16^, band rejection filters^17^, radiating antennas^18^,^19^ and some active devices have been produced successfully based on spoof SPPs^12^,^20^, and it is evident that they will benefit to circuit integration and even system integration in view of the excellence of miniaturization and breaking the challenge of signal integrity^12^,^14^.

Split-ring resonators (SRRs) were originally put forward for their capability to the synthesis of metamaterials with negative effective permeability^21^,^22^,^23^. CSRRs are a kind of defected ground structures by etching SRR elements on the ground plane^24^,^25^,^26^. Regarded as a new type of metamaterial resonators, it has been shown that CSRRs have also negative permittivity^27^. Microstrip SRRs can be considered as the magnetic dipole resonators excited by axial magnetic field, and microstrip CSRRs can relatively be considered as the electric dipole resonators excited by axial electric field^28^,^29^. For inhibiting signal propagation in the vicinity of their resonance frequency, both SRRs and CSRRs are widely used for the design of the miniature microwave devices such as filters, antennas and couplers^29^,^30^,^31^,^32^.

In this article, we propose several band-rejection filters through etching CSRR elements on the SPP structure. The spoof SPP waveguide makes use of double-side or single-side corrugated strips to produce tighter electromagnetic field confinement and smaller propagating wavelength. In order to connect the measured system, coplanar waveguide (CPW) is employed to feed the SPP structures. Two transition sections serve as a bridge between the SPP waveguide and CPW for smooth conversion. SRRs are etched on the metal part of the corrugated strips, coming into being CSRRs. The electric field component perpendicular to the surface of metal can excite CSRRs for inhibiting signal propagation in the vicinity of their resonance frequency. Numerical and experimental results are presented to confirm the good filtering performance of the whole structure. The design method makes the model structure simpler and more compact, and the features of filtering SPP waves are significant for the follow-up developments of plasmonic integrated circuits and systems.

## Results

**Figure 1:**
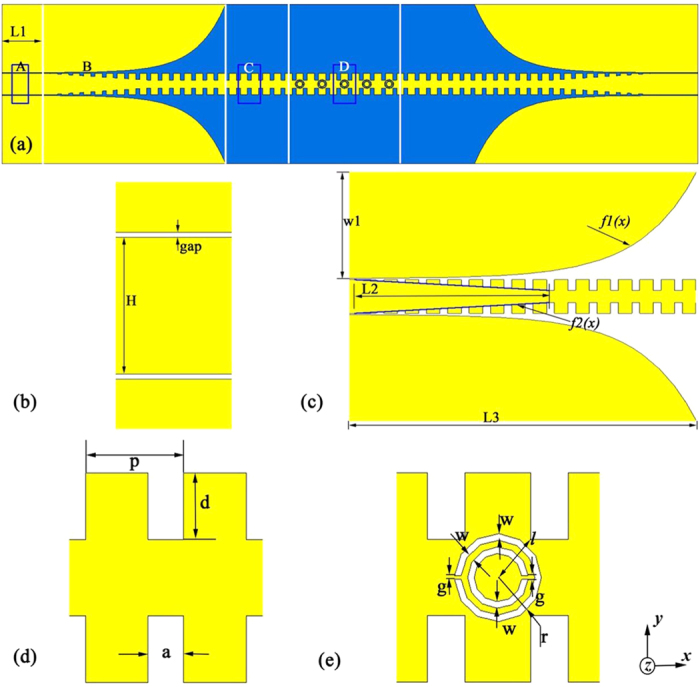
Schematic pictures of the filter by etching SRR elements on the SPP transmission line. (**a**) The overview of the filter. (**b**) The CPW section. (**c**) The transition section with gradient grooves and two flaring grounds. (**d**) The double-side unit cell of the spoof SPP waveguide. (**e**) The CSRR section. All insets show the detailed parameters.

The proposed band-rejection filter is illustrated in Fig. 1, containing the double-side corrugated strips, CSRRs, CPWs and two transition sections, in which the subfigures (b–e) correspond to parts A–D of the whole structure shown in Fig. 1(a), respectively. The designed filter circuit is printed on a thin and flexible dielectric film, F4B, whose relative permittivity is 2.65 (1 + 0.003 i) and thickness is 0.17 mm. The annealed copper (with electric conductivity *σ *= 5.8 e + 007 S/m) is used as metal layers with thickness of 0.018 mm.

For facilitating the experiment with standard SMA connectors, at the end of the filter circuit shown in Fig. 1(b), we design two CPWs to feed the guided-wave energies or receive the spoof SPP signals. Parameters *H* = 11 mm, *gap* = 0.24 mm, and *L*_1_ =20 mm for CPWs are chosen to get 50 Ω input and output impedance. Figure 1(c) depicts a transition section between CPW and the double-side corrugated metallic strip with high efficiency, which has been demonstrated in ref. 13. The transition section containing gradient grooves and two flaring grounds symmetrical to horizontal axis are designed for impedance and momentum matching. The gradient grooves are linear, whose expression is *f*_2_(*x*). The starting and ending points of *f*_2_(*x*) respectively are the highest and the lowest points of the groove, and thus the slope of *f*_2_(*x*) is *L*_*2*_/*d*, where *d* = 3.5 mm refers to the depth of groove and *L*_2_ = 50 mm. The curve of the flaring ground is an exponential function 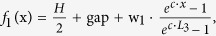 in which the x axis is assumed to be the central line of the double-side corrugated strip and the y axis is located at the left starting side of part B. In the formula, parameters *L*_3_ = 90 mm and w_1_ = 35 mm represent the length and width of the flaring ground, and the degree of the bending curve *f*_1_(*x*) is *c* = 0.07.

In the main transmission section, we adopt a typical configuration of spoof SPP waveguide, a double-side corrugated strip, whose unit cell is displayed in Fig. 1(d). The period of unit arrangement is *p* = 5.5 mm, and the width of groove is *a* = 2 mm. The length of the whole structure is 342.5 mm. The basic principle of the composition of SRRs is two concentric regular polygons or circular ring with opposite splits. Since the symmetry of the double-side corrugation will result in the symmetrical plasmonic modes, SRRs with circular rings are chosen to reach the symmetrically coupling field. As shown in Fig. 1(e), circular SRRs are etched on the metal parts of the corrugated strips, in which the circular-ring center is located at the center of the metal between two grooves. For the integrity of the CSRR structure, the radius of outer ring r should be less than the length between the edge of the groove and the center of the unit cell 
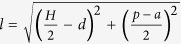
, in case that SRR is too large to be etched on the metal part of corrugation. The ring width and split width are chosen as w = 0.35 mm and g = 0.15 mm, respectively.

**Figure 2:**
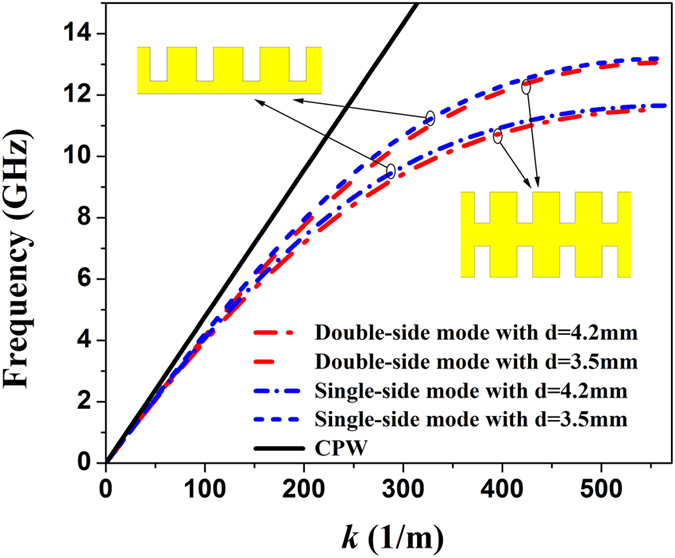
Dispersion diagrams of the single-side (blue lines) and double-side (red lines) SPP waveguides with different groove depths.

The Eigen-mode solver of commercial software, the CST Microwave Studio, is applied to calculate the dispersion curves of the SPP waveguide, comprehending propagation characteristics. In the simulation course, the boundary conditions are periodic in the *x* axis, and electric walls in both *y* and *z* axes, and the distance between the boundary and unit cell is 50 mm in *y* and *z* axes. Figure 2 depicts the dispersion curves of single-side (blue lines) and double-side (red lines) SPP waveguide. We clearly observe that the curves of both modes exhibit SPP-like behaviors, and the cutoff frequency of the double-side corrugation is slightly lower than that of the single-side corrugation with the same groove depth *d*. Furthermore, the dispersion curves of the spoof SPP waveguides deviate gradually from the dispersion curve of CPW marked in black line when the propagation vector *k* grows up, and they asymptotically approach to the lower cutoff frequencies when increasing the groove depth. For spoof SPP waves, the incident energy will be totally reflected when the working frequency is higher than the cutoff frequency because of the physical intermodal coupling between the forward and backward modes around the cutoff frequency^34^. Meanwhile, it implies that both single- and double-side corrugated metallic strips support spoof SPP electromagnetic waves.

**Figure 3:**
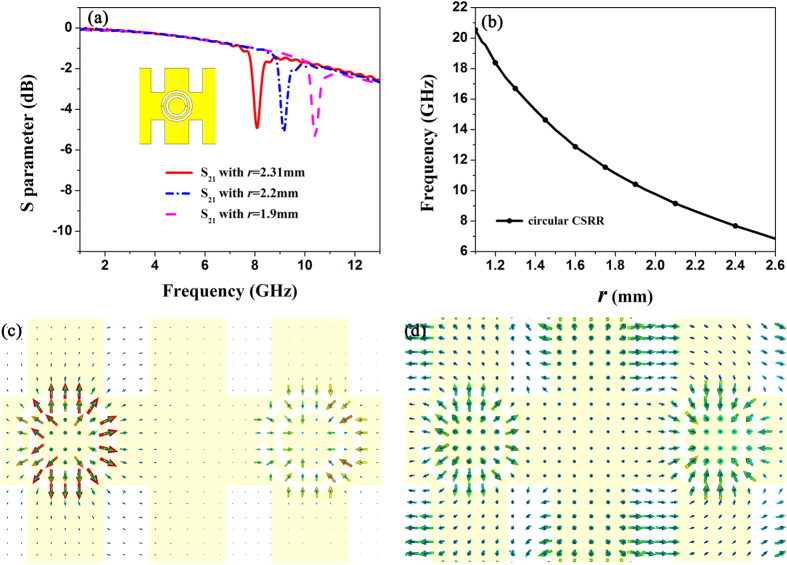
(**a**) The simulated transmission coefficients (S_21_) of the circular CSRRs with different ring radii. (**b**) The simulated relationship between resonant frequency of the circular CSRR and ring radius *r*. (**c,d**) The electric-field distributions of CSRR part with *r* = 2.31 mm at the resonant frequencies 8.15 GHz (**c**), and 9.5 GHz (**d**) within the propagating band.

Using the CST Microwave Studio, we further employ the time-domain solver to simulate the transmission coefficients of the circular CSRRs with different sizes for better apprehending the rejection property. Because CSRRs are considered as electric dipole resonators excited by axial electric field, the boundary conditions are electric wall in the *z* axis and magnetic wall in the *y* axis for guaranteeing the same working environment of CSRRs. Two ports are placed on both ends of the structure with open boundary in the *x* axis. As shown in transmission coefficients in Fig. 3(a), we easily discover that there are three resonance frequencies 8.15, 9.15, and 10.41 GHz when we change the values of the radius as *r *= 2.31, 2.2, and 1.9 mm in the range from 1 to 13 GHz. From Fig. 3(b), we clearly see the simulated relationship between resonant frequency of the circular CSRR and ring radius *r*. In other words, it is visualized and lucid to find that the resonance frequency of CSRR decreases with the increase of the ring radius *r*. Meanwhile, we notice that the CSRR etched on the corrugated metallic structure will reduce the energy transmission. To confirm the insulation of CSRRs, we compare the near-field distributions with *r *= 2.31 mm at the resonant frequency 8.15 GHz and 9.5 GHz within the propagating band. In Fig. 3(c), we notice that the fields are mainly oscillated around the first CSRR, leading to terminated wave propagation at the resonant frequency. However, Fig. 3(d) depicts the near-field distributions propagating without attenuation at 9.5 GHz.

**Figure 4:**
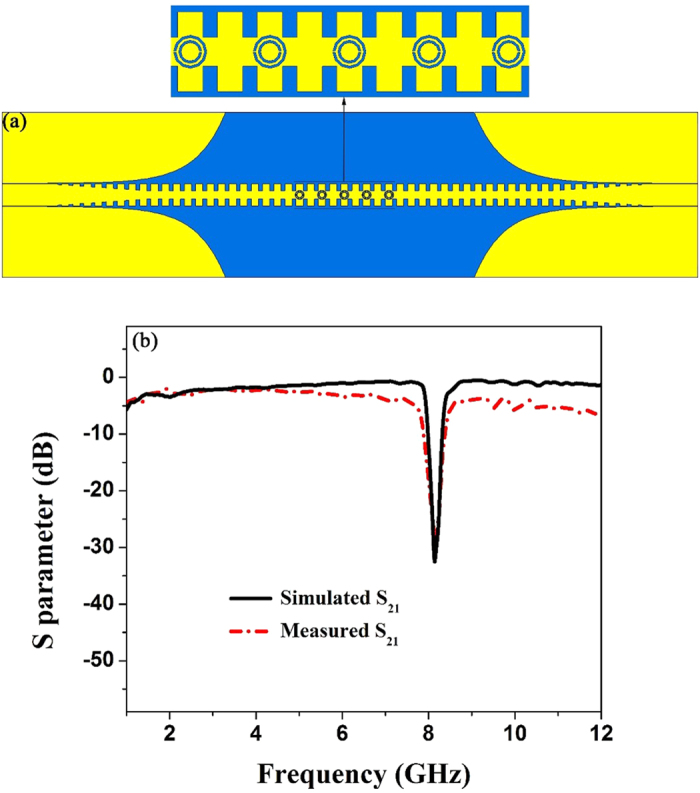
(**a**) The simulation mode of the single-frequency rejection SPP waveguide. (**b**) The simulated and measured transmission coefficients (S_21_) of the fabricated sample.

To study the operation performance of the SPP waveguide etched CSRRs, we fabricate a prototype illustrated in Fig. 4(a), where the enlarged inset shows the details of CSRRs. Five identical CSRRs with *r *= 2.31 mm are etched in the center of the spoof SPP transmission line made of double-side corrugated strips. For measurement, two SMA connectors are welded on both ends of the fabricated sample, and we use two 50 Ω coaxial lines to connect the standard SMA connectors with the Agilent Vector Network Analyzer (VNA, N5230C). From the dispersion curve of double-side mode with *d* = 3.5 mm in Fig. 2(a), we observe that the cutoff frequency of the spoof SPP waveguide adopted in this paper is 13.07 GHz, so that the incident energy will be totally reflected when the working frequency is higher than 13.07 GHz. Figure 4(b) illustrates the simulated and measured transmission coefficients S_21_, respectively, at the propagating frequencies from 1 to 12 GHz lower than the cutoff frequency 13.07 GHz. It is clearly observed that the measured results have a small discrepancy with the simulations, which is mainly caused by the machining tolerance, loss of the coaxial cable, and influence of the test environment in the experiment. From the transmission coefficients, we note that S_21_ is less than −30 dB at 8.15 GHz and the maximum S_21_ can reach −0.5 dB within the −3 dB bandwidth. As shown in Fig. 3(a), the frequency 8.15 GHz is the resonant frequency with *r *= 2.31 mm, so that it is proved that the electric field component perpendicular to the metal surface can excite CSRRs. Meanwhile, the simulation results confirm the excellent filtering characteristics such as small insertion loss, significant rejection, high transmission efficiency, and great square ration. The measured results further confirm the practicability of the proposed structure.

**Figure 5:**
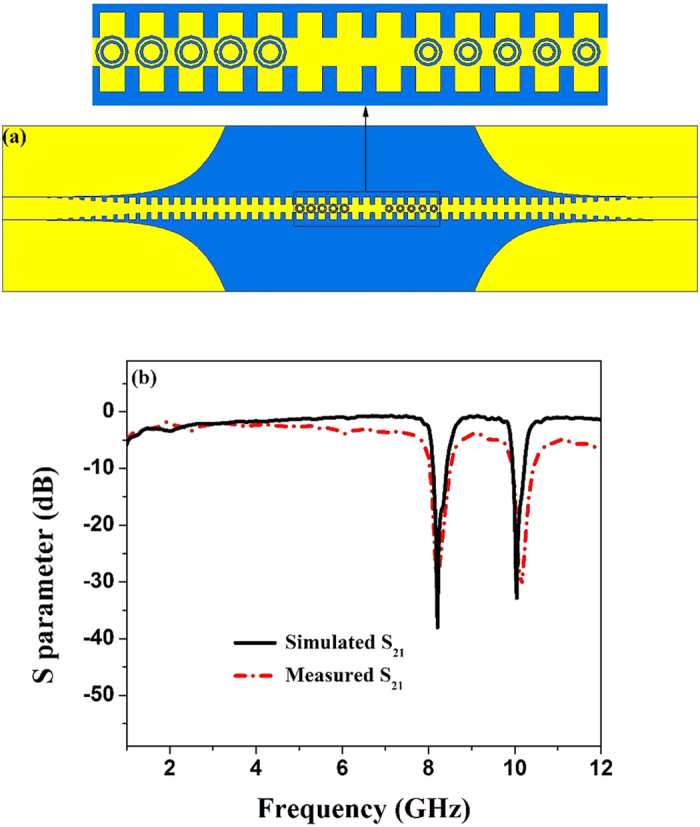
(**a**) The simulation mode of the double-frequency rejection SPP waveguide. (**b**) The simulated and measured transmission coefficients (S_21_) of the sample.

Owing to the maneuverability of the CSRR resonant frequencies with different circular-ring radii, we fabricate a double-frequency rejection SPP waveguide by using two kinds of CSRRs. As shown in Fig. 5(a), there are five CSRRs with circle radius *r *= 2.30 mm and other five CSRRs with circle radius *r *= 1.95 mm. Such ten CSRRs are etched on the central metal part of the corrugated strip, and the interval between the two kinds of CSRRs is three times of the structure period. From the S scattering parameters of CSRRs with different circle radii, we notice that the resonant frequency is higher when the radius diminish in size. It is obvious that for CSRRs, there are two different resonant frequencies when *r *= 2.30 mm and *r* = 1.95 mm, respectively. As the simulated and measured results illustrated in Fig. 5(b), we confirm that the proposed structure provides the excellent rejections at 8.21 GHz and 10.04 GHz. The simulated transmission coefficients at both 8.21 GHz and 10.04 GHz are less than −30 dB. On the basis of the performance of the tunable resonant frequency of CSRR, it is convenient to fabricate the multiple-frequency rejection filters and integrated circuits.

**Figure 6:**
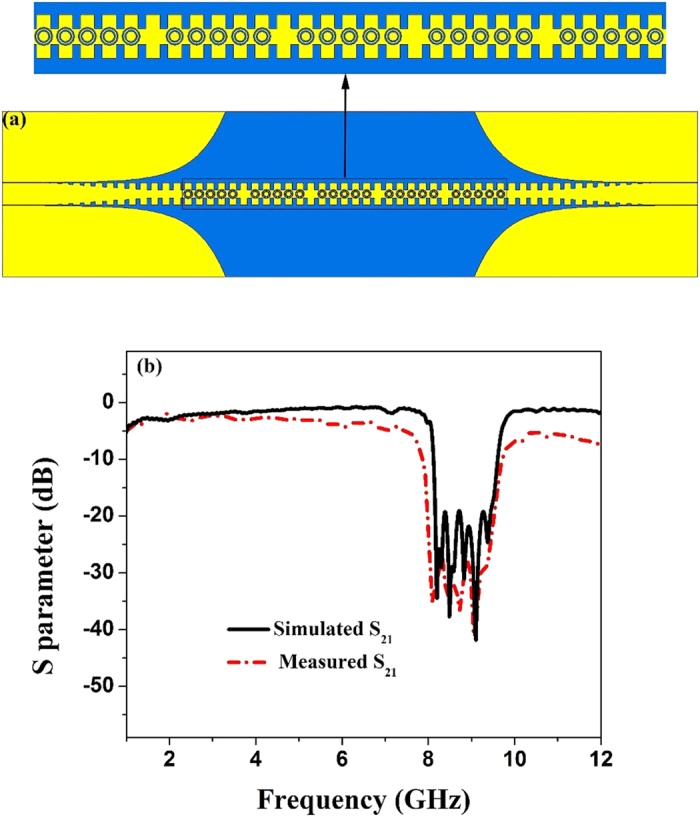
(**a**) The simulation mode of the broad band-stop SPP waveguide. (**b**) The simulated and measured transmission coefficients (S_21_) of the sample.

Since CSRRs always work in narrow frequency band, a small amount of CSRRs cannot achieve a broadband-frequency rejection. In other words, several kinds of CSRRs with different sizes can realize multi-band filtering. By adjusting the size of the structure, the adjacent resonant frequency points are close to each other, so that the broadband filtering effect is reached. Therefore, we use a series of CSRRs with different circle radii to fabricate the broad band-stop filter, as shown in Fig. 6(a). By optimizing the size of circle ring, we choose twenty-five etched CSRRs including five CSRRs with circle radius *r *= 2.32 mm, five CSRRs with circle radius *r *= 2.25 mm, five CSRRs with circle radius *r *= 2.19 mm, five CSRRs with circle radius *r *= 2.13 mm, and five CSRRs with circle radius *r *= 2.07 mm. Each different kind of circular ring set is separated from the others by a unit period of corrugation. The filtering performance of the broad band-stop is authenticated through Fig. 6(b), illustrating the simulated and measured transmission coefficients. We note a remarkable stop band from 8.11 GHz to 9.61 GHz, and the simulated S_21_ of the stop band is less than −20 dB. It is clearly clarified the property of frequency rejection in a broad band on the proposed SPP waveguide.

**Figure 7:**
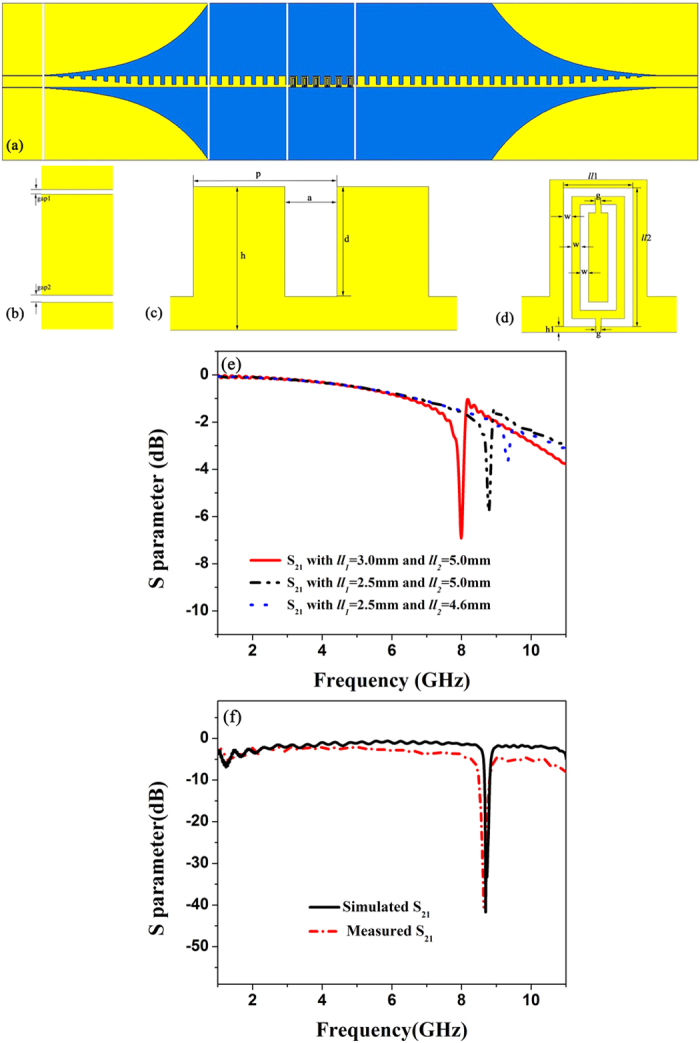
(**a**) The simulation model of the single-side filtering SPP waveguide. (**b**) CPW section. (**c**) The single-side unit cell. (**d**) The rectangular CSRR section. (**e**) The simulated transmission coefficients (S_21_) of CSRRs with different sizes. (**f**) The simulated and measured S_21_ of the sample.

It has been confirmed that both single-side and double-side corrugate metallic strips can support the propagation of spoof SPPs^33^. Figure 7(c) displays the unit cell of the single-side SPP waveguide with the width *h *= 5.5 mm. The period of single-side unit arrangement is *p *= 5.5 mm, and the width and depth of groove are *a *= 2.2 mm and d = 4.2 mm, respectively. Due to the asymmetry of the single-side corrugated strip, we choose two different gaps *gap1 *= 0.15 mm and *gap2 *= 0.2 mm to describe CPW, as illustrated in Fig. 7(b). The gradient grooves are linear, whose slop is *L*_2_/*d* where *L*_2_ = 45 mm, indicating the length of the gradient grooves. *L*_3_ = 80 mm represents the length of the flaring ground, and the degree of the bending curve part of flaring ground is *c *= 0.04. The length of whole structure is 337 mm, and other parameters that are not specified are the same as those in the numerical mode shown in Fig. 1. In virtue of the asymmetric SPP waves resulting from the single-side corrugation and the size of SRR to be etched on the metal part of the waveguide, we adopt rectangular CSRRs which are not symmetric with the y axis for coupling the asymmetric SPP waves, in which the rectangular CSRRs have smaller relative sizes for achieving the compact structure. As illustrated in Fig. 7(d), the length and width of the outer rectangle are denoted as *ll*_1_ = 2.5 mm and *ll*_2_ = 5.0 mm, respectively, and the size *ll*_1_ × *ll*_2_ should be smaller than (p-a) × h for integrity of the rectangular CSRRs. The rectangular CSRRs are located at the position of *h*_1_ = 0.2 mm away from the bottom. We use *w* = 0.3 mm and g = 0.2 mm to depict the ring width and split width, respectively.

In order to understand the performance of the rectangular CSRR, we firstly simulate the transmission coefficient of a single rectangular CSRR. Figure 7(e) depicts three transmission coefficients with three sizes *ll*_1_ × *ll*_2_ = 3.0 × 5.0, *ll*_1_ × *ll*_2_ = 2.5 × 5.0, and *ll*_1_ × *ll*_2_ = 2.5 × 4.6 mm^2^. It is observed that the resonance frequencies 8.0, 8.79, and 9.32 GHz of rectangular CSRRs increase as the size decreases. Then, the simulation mode of the single-side waveguide etched with six rectangular SRRs is fabricated, as given in Fig. 7(a). From the simulated and measured S scattering parameters of the filter illustrated in Fig. 7(f), we note a conspicuous stop band at 8.74 GHz where the transmission coefficients are less than −30 dB. Because the cutoff frequency of the single-side SPP waveguides with *d* = 4.2 mm adopted in this paper is 11.66 GHz, as shown in Fig. 2, we choose the propagating frequencies from 1 to 11 GHz. The simulated results confirm that the single-side SPP waveguide etched with the rectangular SRRs also have the excellent filtering characteristics and this structure can play an important role to develop plasmonic integrated circuits.

## Discussion

**Figure 8:**
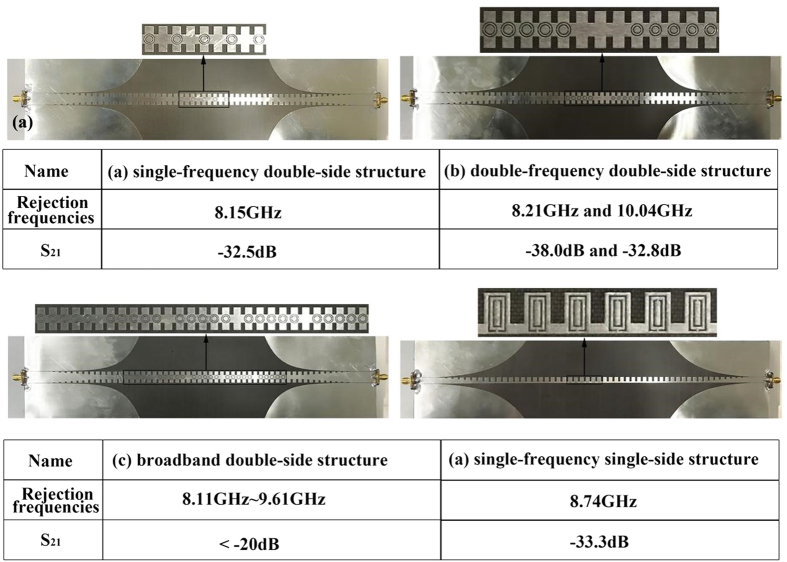
The photograph of (**a**) single-frequency double-side structure, (**b**) double-frequency double-side structure, (**c**) broadband double-side structure, and (**d**) single-frequency single-side structure. The detailed geometry of each structure is given in each photograph.

**Figure 9:**
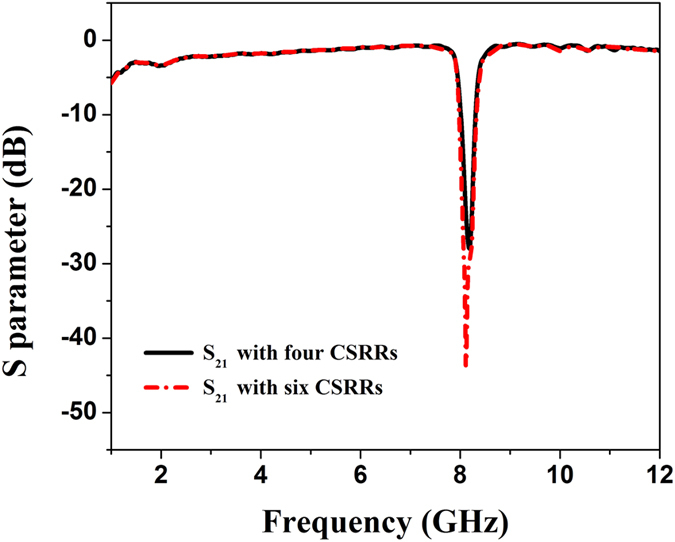
The simulated transmission coefficients (S_21_) of the single-frequency double-side structure with four CSRRs (black line) and six CSRRs (red line).

From both simulation and measurement results in shown in Figs 4(b), 5(b), 6(b) and 7(f), we find that the proposed structures have highly efficient filtering performance. Figure 8(a–d) illustrate the photographs of single-frequency double-side, double- frequency double-side, broadband double-side, and single-frequency single-side structures, respectively. The insert in each photograph shows the detailed geometry for the convenient comprehension. Compared with previous rejection filters based on spoof SPP waveguide^17^, the rejection bandwidth has been increased for the broadband rejection filter, and the isolation of rejection frequencies is improved significantly, and even reaches to −38 dB for the single-frequency rejection filters. Increasing the numbers of CSRRs can increase the isolation, as shown in Fig. 9 for the comparison between four and six CSRRs. On the other hand, the proposed structures are so simple and compact that they do not take up extra space. Because the single-side and double-side structures are manageable alone, it is convenient to control the signal filtering of each branch for SPP integrated circuits and systems.

**Figure 10:**
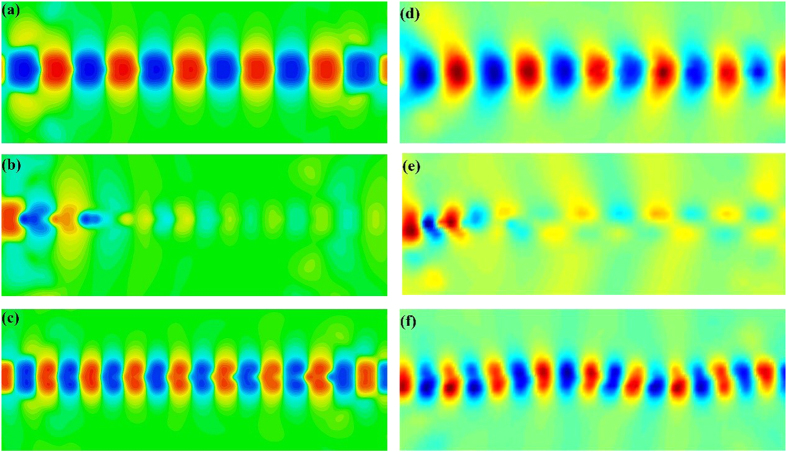
The simulated and measured near-electric-field distributions on the *x–y* plane which is 4 mm above the dielectric substrate at different frequencies. The simulated results at (**a**) 7 GHz (the pass band), (**b**) 8.6 GHz (the resonant frequency), and (**c**) 10 GHz (the pass band). The measured results at (**d**) 7 GHz (the pass band), (**e**) 8.6 GHz (the resonant frequency), and (**f**) 10 GHz (the pass band).

For obtaining the physical insight into the SPP waveguide etched with CSRRs, we study the near-field electric distributions of *z*-components on the *x*–*y* plane, which is 4 mm above the dielectric substrate. As example, we chose the broadband double-side sample for near-field simulations and measurements. The field monitor for electric fields is set up at some frequencies, and the simulated field distributions are illustrated in Fig. 10(a–c). At the frequency 8.6 GHz at the rejection band, we note that the electric-field distribution is interrupted at the location of CSRRs, as demonstrated in Fig. 10(b). Because the corrugated metallic strip generates the electric-field component perpendicular to the SPP waveguide, CSRRs can be excited to the resonant state at the resonant frequency.

However, at 7 GHz and 10 GHz in the pass band, we observe that the SPP waves are propagating through the corrugated strip and CSRRs with very small loss, as shown in Fig. 10(a,c). We also notice that the transition sections can effectively realize the coupling and conversion between the spatial guide waves and spoof SPPs in the filtering circuit. Figure 10(d–f) depict the relevant results of the near-field electric distributions measured by a home-made near-field scanning system, which have very good agreements with the numerical simulations. Both the simulated and measured results intuitively provide a proof to corroborate the great filtering performance of the transmission device for the plasmonic integrated circuits at microwave frequencies.

## Conclusion

By mean of the circular and rectangular SRRs etched on the SPP waveguides using double-side and single-side corrugated metallic strips, we have presented several novel compact band-rejection filters to develop highly efficient and controllable rejection-band plasmonic circuits. In virtue of the highly confined electromagnetic waves of SPPs and smooth conversion of the transition sections, the proposed filters have excellent transmission efficiency and low loss. Meanwhile, owing to the fact that the electric field component perpendicular to the metallic surface will excite CSRRs, there are some signal propagation rejections at special designed resonance frequencies. Based on this principle, we have designed the single-frequency rejection filters, double-frequency rejection filters, and broadband rejection filters using single-side and double-side SPP waveguides. All such structures have been manufactured for experimentally verifying the marvelous capability. Both simulation and measurement results have demonstrated the highly efficient filtering performance. The proposed tunable band-rejection filters provide potentials to promote the development of plasmonic functional devices and integrated circuits in both microwave and terahertz frequencies.

## Methods

Numerical simulations are performed by commercial software, the CST Microwave Studio, whose eigen-mode solver and time-domain solver are employed to calculate the dispersion relation and S parameters, respectively. A 0.17-mm thin and flexible dielectric film F4B with the relative permittivity 2.65 (1 + 0.003 i) is used to fabricate the experimental filters. The metal layers with thickness 0.018 mm are adopted as a kind of annealed copper (electric conductivity *σ *= 5.8 e + 007 S/m). For experiments, we utilize Agilent Vector Network Analyzer (VNA, N5230C) to measure S parameters of the fabricated filters. A home-made near-field scanning system is used to measure the near electric-field distributions.

## Additional Information

**How to cite this article**: Zhang, Q. *et al.* A series of compact rejection filters based on the interaction between spoof SPPs and CSRRs. *Sci. Rep.* **6**, 28256; doi: 10.1038/srep28256 (2016).
